# Risks of Heat Illness in Athletes With Spinal Cord Injury: Current Evidence and Needs

**DOI:** 10.3389/fspor.2019.00068

**Published:** 2020-01-10

**Authors:** Yang Zhang, Phillip A. Bishop

**Affiliations:** ^1^Independent Researcher, Jiaxing, China; ^2^Department of Kinesiology, The University of Alabama, Tuscaloosa, AL, United States

**Keywords:** paralympic, paraplegia, tetraplegia, thermoregulation, hyperthermia

It has been well-documented that spinal cord injury (SCI), especially with resultant tetraplegia or high level paraplegia (T6 and above) (Price and Campbell, 2003), leads to disrupted somatic, sensory, and autonomic functions below the level of lesion (Cruz and Blauwet, 2018; Walter and Krassioukov, 2018), thereby compromising the heat dissipation mechanisms (Price and Trbovich, 2018). The current consensus when it comes to exercise and sports in the heat is that athletes with SCI, multiple sclerosis, or cerebral palsy (another neuromuscular disorder affecting thermoregulation) are more susceptible to hyperthermia than able-bodied athletes (Lepretre et al., 2016). The purpose of this review was to examine the published evidence to assess the risk of heat injury during competition and training in SCI athletes and use this information to provide a basis for improved protection for these sports.

Empirical evidence to date, however, is notably limited regarding the assumed risks of heat injury among this cohort (Price, 2016), which could be due to a very small incidence count, lack of resources, or failures to collect data (Trbovich et al., 2019). Grobler et al. (2019), for the first time, reported accurate medical records (level III evidence) during the 2015 IPC Athletics World Championships held in the heat (venue wet-bulb globe temperature 24.6–36.0°C). According to their field data, not only was the incidence rate of all illnesses (37.6 per 1,000 para athletes) low in comparison to that from outdoor IAAF World Championships between 2009 and 2017 (50.2 per 1,000 able-bodied athletes) (Edouard et al., 2019), but it was also explicitly low in heat illness (seven recorded cases of heat illness, of which there was only one case of athlete with SCI).

The discrepancy between hypothesis and observation is that the cause of the elevated body temperature (which ultimately leads to heat illness) during the time course of exercise and sports is not solely attributed to the body's heat dissipation mechanisms. Rather, the absolute heat production rates and the capacity of the body to transport heat and the environment to absorb the heat generated by the body are the real issues among this cohort. This is akin to what economists call an “identification problem.” For instance, Olympic-caliber marathoners reached a VO_2max_ of 79.6 ml·kg^−1^·min^−1^, and they ran at an average of 89.7% VO_2max_ (resultant oxygen consumption ~71 ml·kg^−1^·min^−1^) during a 10-km time trial (Billat et al., 2001). In contrast, Paralympic-caliber wheelchair racers with SCI showed considerably lower VO_2max_, 46.4 ml·kg^−1^·min^−1^ as well as a substantially lower percentage of VO_2max_, 73.7% (resultant oxygen consumption ~34 ml·kg^−1^·min^−1^) during a 25-km time trial (Edwards et al., 2018). Furthermore, due to the difference in race velocity, the duration of endurance sports is typically longer among able-bodied events; for example, the average time of Rio 2016 Olympics and Paralympics marathon finishers was Men, 02:22:22 vs. T54 Men, 01:32:14 and Women, 02:43:34 vs. T54 Women, 01:42:04^1^. Therefore, given the same body mass, the metabolic energy expenditure, which is a proximate cause of heat illness, and thereby the cumulative heat production is relatively lower among para athletes.

In terms of competition environments, many para sports, such as wheelchair basketball, are held at indoor stadiums where the venue temperature is typically controlled at 19–21°C. Such temperate environments could partially offset the deleterious effects of the autonomic nervous system related dysfunction in thermoregulation. Certain para athletes participating in outdoor endurance events (e.g., T54 wheelchair athletes competing in 5,000 m, 10,000 m, marathon, and H1–5 para cyclists) could be subject to the negative influence of heat on performance and health when competitions are held in hot and humid environments. However, the nature of these sports allows para athletes to get some thermoregulatory relief in the form of enhanced convection and evaporation from air movement. While Olympic-caliber marathoners ran at a 18.8 km·h^−1^ pace (Billat et al., 2001), Paralympic-caliber wheelchair racers with SCI showed an average race speed of 30.7 km·h^−1^ (Edwards et al., 2018), which resulted in significantly faster air motion from the wheelchair movement relative to the environment.

In the able-bodied population, manipulating air velocities of 10–33.5 km·h^−1^ during 2-h submaximal exercise in the heat (33°C ambient temperature, 59% relative humidity) reduced heat storage by 47–60% compared with a no-wind condition (Saunders et al., 2005). Similarly, combining an air velocity of 9.2 km·h^−1^ and rehydrating 100% of sweat losses during 1-h submaximal exercise in the heat (36°C ambient temperature, 29% relative humidity) reduced heat storage by 41% whilst also maintaining cardiac output compared with control conditions in a group of heat-acclimated healthy males (Mora-Rodriguez et al., 2007). Although these findings from able-bodied persons cannot be directly extrapolated to SCI athletes who show compromised sweating responses (Price and Trbovich, 2018), in particular among athletes with tetraplegia (e.g., >50% reduction in estimated whole body sweat rate for tetraplegia vs. paraplegia) (Price and Campbell, 2003), SCI athletes competing in the outdoor wheelchair 10,000 m, marathon, and paratriathlon could still get thermoregulatory relief from greater air flow, which results in better evaporation. This has been demonstrated in a field study showing that wheelchair athletes (comprised mostly of SCI athletes) displayed significantly a smaller increase in core temperature compared with athletes with a visual impairment (i.e., +0.26°C vs. +1.03°C) during the running segment of a paratriathlon (Stephenson et al., 2019). Therefore, despite the compromised heat dissipation mechanisms, SCI athletes generally carry less than expected accumulated thermal burden as a result of both a relatively lower heat production rate and higher evaporative heat transfer to the environment.

It is worth noting that the ability to maintain core body temperature within normothermia while exercising in the heat would be affected by the varying degrees of sympathetic integrity in this cohort. There is consistent reporting of continual increases in core body temperature during rest (Griggs et al., 2019a) and exercise (Price and Trbovich, 2018) among persons with tetraplegia. For example, competitive wheelchair rugby match play (4 ×8-min quarter; ambient temperature, 18.4–20.9°C; relative humidity, 31.1–45.1%) resulted in 39.3°C core body temperature in elite players with a cervical SCI (C5/6–C7) (Griggs et al., 2017). A popular perception is therefore that athletes with tetraplegia, who exhibit greater disruption of evaporation (sweating) and convection (cutaneous vasodilation), should be prepared with appropriate cooling strategies during exercise in the heat provided they demonstrate heightened thermal strain (Griggs et al., 2019b; Trbovich et al., 2019). While this increase in core body temperature, especially if >39.0°C, could be materially alarming, trained tetraplegic athletes who participate in indoor events (e.g., wheelchair basketball, rugby, and fencing) or outdoor sports (e.g., H1 para-cycling), exhibit no expected higher incidence rate of heat injury. True exertional heat injury is primarily triggered by a pathological elevation of the core body temperature, usually >40.5°C (Casa et al., 2015). Additionally, severe heat injury, including exertional heat stroke, could have resulted from excessive endogenous thermogenesis, leading to widespread muscle necrosis and even organ failure (Rae et al., 2008). To date, none of these unfortunate medical comorbidities have been documented in this cohort. Furthermore, another important catalyst for exertional heat injury is prolonged duration of continuous exercise. Athletes with tetraplegia usually compete in much shorter durations compared with able-bodied endurance events, such as marathons and triathlons, that incur more frequent cases of heat injury. Therefore, despite severe interruption of effector sympathetic pathways in athletes with tetraplegia, the hypothesized higher risks of heat injury (Price, 2016) is not supported by the available epidemiological data.

However, the low incidence rate of illness, including heat-related illness, among para athletes (Grobler et al., 2019) should not be celebrated. No one is immune to heat illness, and para athletes are no exception. Preventive measures and post-incident medical strategies are still needed to ensure overall health, especially if events are held in hot and humid environments. Thermoregulatory data recorded at a paratriathlon (time to completion 54.6–103.9 min) in the heat (33°C ambient temperature, 35–41% relative humidity, 25–27°C water temperature) have revealed that 78.6% of studied para athletes displaying a core body temperature >39.5°C, including 28.6% of para athletes showing a core body temperature >40.0°C (Stephenson et al., 2019). This exertional hyperthermia was further tied to 57% of studied para athletes experiencing self-reported symptoms of heat illness (Stephenson et al., 2019). The field study by Stephenson et al. (2019) also indicated that, although the incidence rate of heat illness may not be officially documented, para athletes participating in endurance sports may very well be experiencing much greater deep body temperatures, and suspected symptoms of heat illness and their consequences may therefore be overlooked by these athletes. Proper education of athletes, coaches, and event organizers about heat injury is thus warranted. Without a well-prepared framework for early recognition, diagnosis, and treatment of heat illness, persistent exertional hyperthermia could lead to thermoregulatory collapse, resulting in heat injury in any type of athlete.

Across a variety of organized sports that SCI athletes are eligible to compete in, wheelchair marathons, triathlons, and tennis usually last over 60 min, making these SCI athletes vulnerable to heat illness in warm/hot weather. Despite this potential health risk, policies regarding safe competition under heat exposure for SCI athletes have only been implemented by the International Tennis Federation (Regulations for Wheelchair Tennis 2019, September 2019)^2^, which still lacks specific guidelines for the risk management of heat illness. In comparison, governing bodies of able-bodied sports, such as National Collegiate Athletic Association^3^ and Australian Open^4^, have established specific heat policies to maximize the safety for sport competition in the heat.

Moreover, it is crucial to understand that SCI athletes display unique patterns of thermoregulatory responses as a consequence of their underlying physiology. Cutaneous temperature sensation has been studied extensively, including in persons with SCI (Price and Trbovich, 2018). In response to the stimulus, nerve impulses regarding the thermal state of the body and the environment are sent to the spinal cord, the major bidirectional connection between the body and the brain, where neural signals are transmitted upwards until they are blocked at the level of the lesion. Reduced afferent input from the insensate portion of the body, especially at those regions (i.e., chest, forearm, hand, finger, and thigh) rich in warm thermoreceptors (Arens and Zhang, 2006), results in altered thermal sensation. Griggs et al. (2019a) presented data showing that active persons with SCI undergoing passive heat exposure (37°C ambient temperature; initially 20% relative humidity, with an increase by 5% every 7 min thereafter), despite displaying warmer mean skin temperature, were not able to perceive the magnitude of thermal strain as measured using thermal sensation. Should athletes present higher levels (tetraplegia vs. paraplegia) of SCI, or completeness (vs. incompleteness) of SCI, the resultant thermal sensation in the heat is expected to be further disrupted.

In summary, abnormal somatic, sensory, and autonomic functions after SCI present significant challenges for these individuals participating in competitive sports. Despite the fact that thermal dysfunction exists, the circumstances of SCI sports competition may largely mitigate any compromise of heat dissipation in these athletes, leading to the low incidence rate of heat illness. For marathon, tennis, and paratriathlon, the nature and distinct risks of heat illness of any para athlete competing in warm to hot environments underline the need for specific guidelines aimed at improving knowledge and medical support for athletes with SCI in particular. Continued research to evaluate heat tolerance among all athletes in all sports is vital to safe sports competition. For the upcoming Tokyo 2020 Paralympics, the environmental conditions are expected to pose a challenge to para athletes' performance and health (Kakamu et al., 2017). Accordingly, a systematic heat policy addressing appropriate preventive measures (Griggs et al., 2015, 2019b), early recognition and correct diagnosis (Epstein and Yanovich, 2019), and effective treatment strategies (Belval et al., 2018) of heat illness would be essential to uphold safe and successful sports participation for this special population.

## Author Contributions

YZ and PB drafted the manuscript and read and approved the submitted version.

### Conflict of Interest

The authors declare that the research was conducted in the absence of any commercial or financial relationships that could be construed as a potential conflict of interest.

## Footnotes

^1^Results retrieved from Rio 2016 Summer Olympics Men's and Women's Marathon and Rio 2016 Paralympic Games Men's and Women's Marathon—T54. Available online at: https://www.olympic.org/rio-2016/athletics/marathon-men, https://www.olympic.org/rio-2016/athletics/marathon-women, https://www.paralympic.org/rio-2016/schedule-results/info-live-results/rio-2016/eng/at/engat_athletics-results-men-s-marathon-t54-1-01.htm, https://www.paralympic.org/rio-2016/schedule-results/info-live-results/rio-2016/eng/at/engat_athletics-results-women-s-marathon-t54-1-01.htm (accessed October 9, 2019).

^2^Regulations for Wheelchair Tennis 2019 (2019). Available online at: https://www.itftennis.com/media/309868/309868.pdf (accessed September 26, 2019).

^3^Hydration breaks approved in men's and women's soccer (2019). Available online at: http://www.ncaa.org/about/resources/media-center/news/hydration-breaks-approved-men-s-and-women-s-soccer (accessed October 15, 2019).

^4^Extreme Heat Policy (2019). Available online at: https://ausopen.com/articles/news/ao-heat-stress-scale-grand-slam-first (accessed October 15, 2019).
